# Protective effects of colorless carotenoid precursors against UV-induced lipid oxidation in liposomes compared to lycopene

**DOI:** 10.1038/s41598-026-53721-y

**Published:** 2026-05-20

**Authors:** Anna Heidrich, Volker Böhm

**Affiliations:** Institute of Nutritional Sciences, Friedrich Schiller University Jena, 07743 Jena, Germany

**Keywords:** Phytoene, Phytofluene, Antioxidant activity, Antioxidant degradation, Tomato extract, UV-radiation, Biochemistry, Biophysics, Chemical biology, Chemistry, Plant sciences

## Abstract

Consuming tomato-extracts can reduce the risk of developing chronic diseases. As lycopene supplementation alone had a weaker impact, the protective effect may be influenced by the colorless carotenoids phytoene and phytofluene. To obtain more insights into the effect of tomato-extracts, the three carotenoids were separately investigated. UV-C-, UV-B- and UV-A-irradiated liposomes containing the antioxidants were analyzed with the thiobarbituric acid-assay coupled with HPLC-DAD, using malondialdehyde as biomarker for lipid oxidation. Antioxidant degradation through UV-irradiation was analyzed by HPLC-DAD. Phytoene had a similar protective effect of 27–34% under UV-C- and UV-B-radiation compared to lycopene. But under UV-A-radiation no effect was observed, and lycopene showed the highest effect. Phytofluene, only examined under UV-B- and UV-A-radiation, showed just pro-oxidative effects. After UV-B-radiation, 13% phytofluene were left, and it completely degraded under UV-A-radiation. Phytofluene’s instability in the presence of oxygen and its high ratio of cis-isomers might have led to these results. The protective effect of phytoene compared to lycopene is due to its higher absorption of UV-C- and UV-B-radiation. In contrast, lycopene maintains its effect under UV-C-radiation because of its high number of conjugated double bonds, whereby radicals are better resonance-stabilized.

## Introduction

**Fig. 1:**
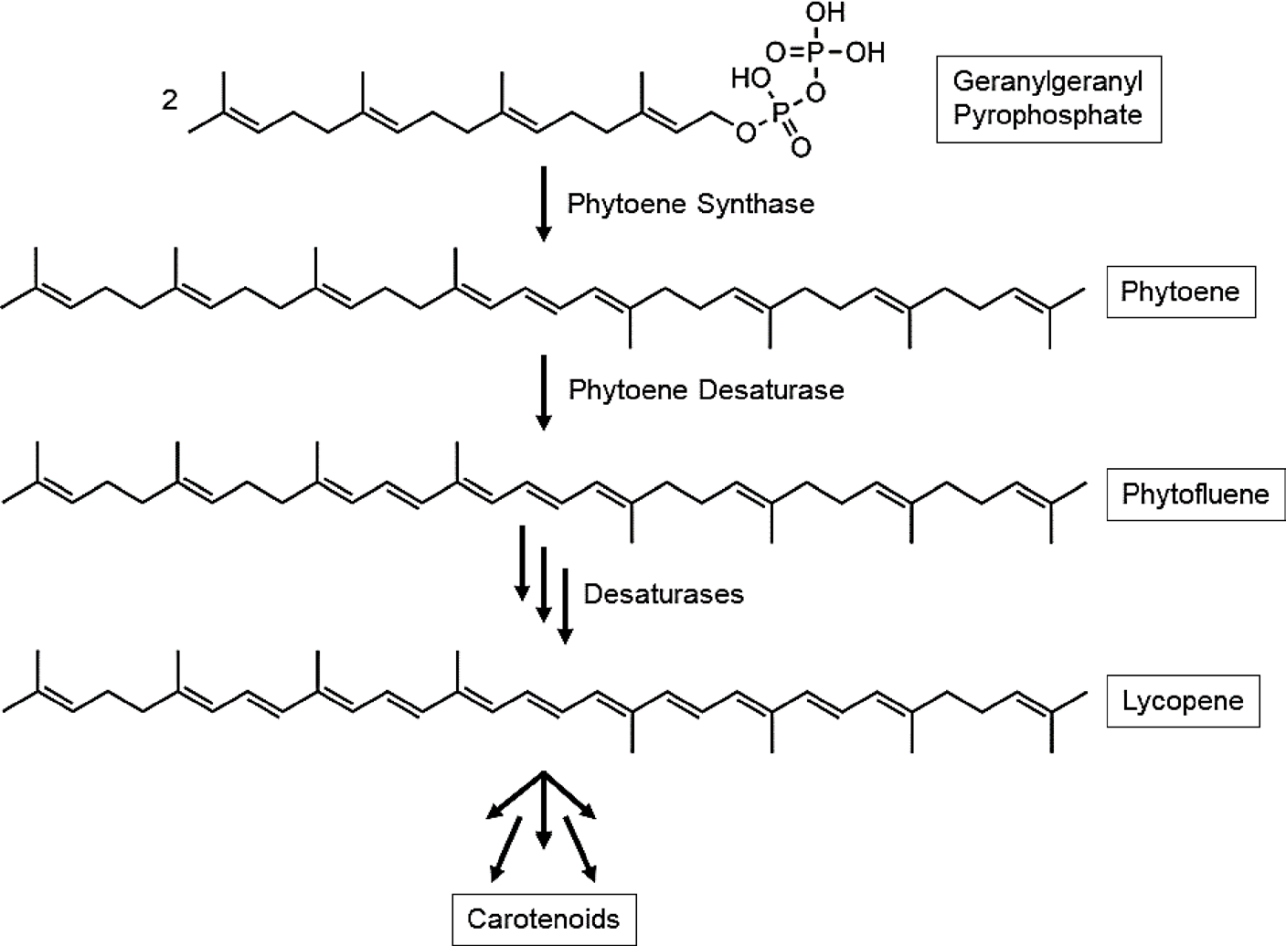
Carotinoid-Synthesis^5^.

Each person consumed 7.36 kg of tomatoes on average in 2025 worldwide^1^. Compared to other vegetables, the tomato is one of the most widely eaten fruits^2,3^. Tomato products are utilized in numerous studies to analyze the protective effects of the bioactive plant substances, especially the carotenoids, against cancer and cardiovascular diseases^4^. Initially, the protective effect of tomatoes was attributed to the carotenoid lycopene. Although lycopene is the most abundant carotenoid in tomatoes, the protective effect of tomatoes does not appear to be exclusively due to lycopene, but also due to the colorless carotenoid precursors phytoene and phytofluene^5–7^. For example, the study by Aust et al., 2005, showed that the minimum erythema dose (MED) increased significantly in the groups consuming tomato extracts (Lyc-o Mato^®^, LycoRed, Beer Sheva, Israel) compared to the group ingesting pure synthetic lycopene, after 12 weeks of supplementation. For the group which were given just lycopene, the MED did not increase significantly^8^. Phytoene is formed during carotenoid synthesis, which occurs exclusively in photosynthetic organisms and individual fungi and bacteria, from two geranylgeranyl pyrophosphates by phytoene synthase^9,10^. Then, a desaturase produces another colorless carotenoid, the phytofluene. From this, lycopene can be synthesized by further desaturases, which in turn can be used to form further carotenoids (Fig. 1)^9,11^.

**Fig. 2:**
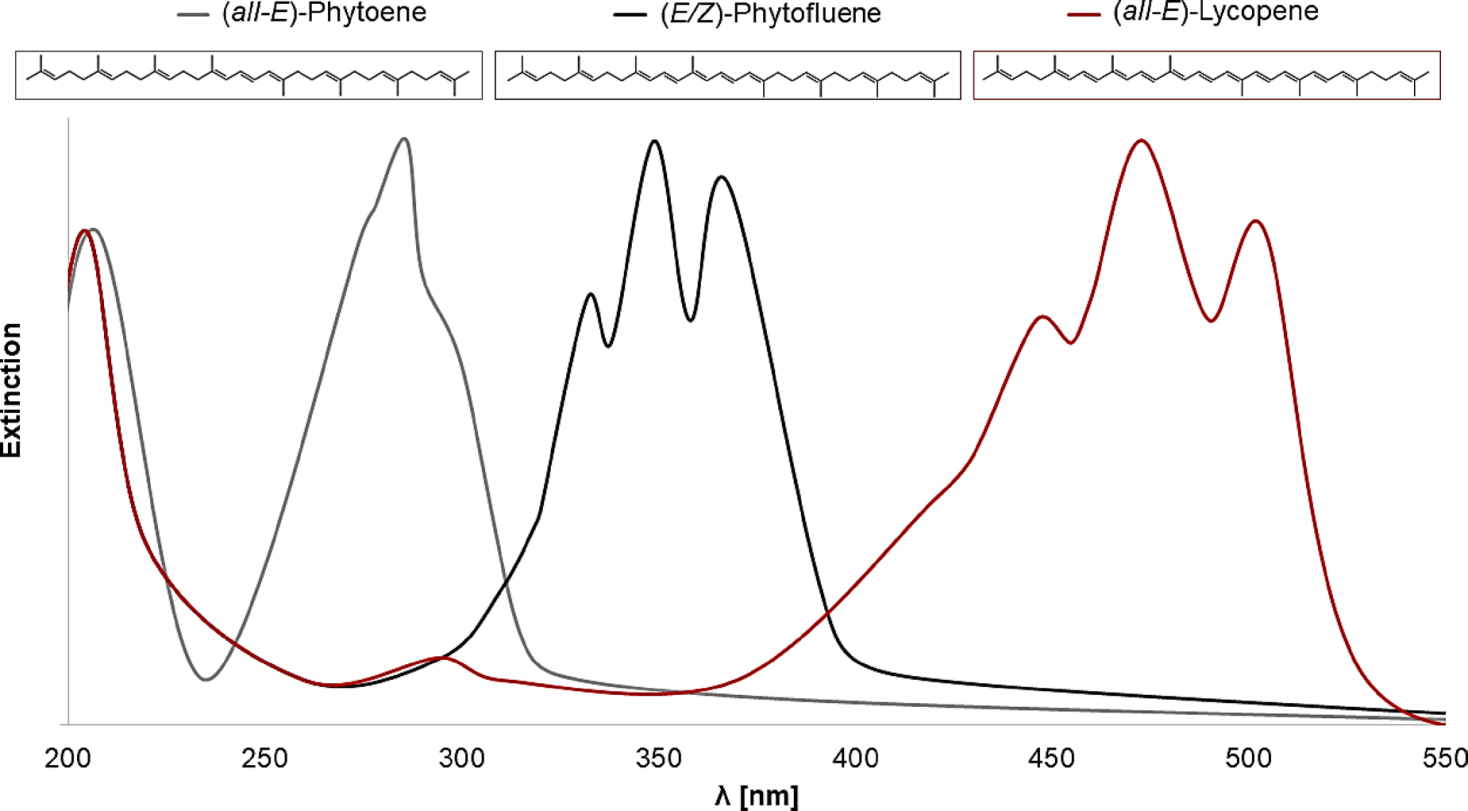
Absorption spectra of the carotenoids phytoene, phytofluene and lycopene.

Phytoene, phytofluene and lycopene are therefore all tetraterpenes, but they differ in their number of double bonds. With three conjugated double bonds, phytoene has the fewest of a total of nine double bonds. For this reason, the absorption maximum is at 286 nm. Phytofluene has five conjugated double bonds out of a total of ten double bonds and therefore absorbs lower-energy light with a maximum at 347 nm. Lycopene has eleven conjugated double bonds out of a total of thirteen double bonds. The number of double bonds and especially the number of conjugated double bonds is decisive for the light absorption and therefore its color and reactivity^10^. The carotenoids appear colored when they have seven or more conjugated double bonds. Due to the high number of conjugated double bonds of lycopene, the maximum absorption is at 474 nm^10,12^. The absorption spectra are shown in detail in Fig. 2.

Since phytoene and phytofluene are precursors of carotenoids and found in carotenoid-rich foods, their intake should not be neglected. In many carotenoid-rich foods, such as apricots and papayas, carotenoid precursors are the carotenoids found in the second-highest concentration^7,13^. According to Biehler et al., 2012, who analyzed the carotenoid intake in the Luxembourg population, phytoene is ingested at a carotenoid content of 12% already after the more known α- and β-carotene. Luxembourg consumes relatively few tomato products compared to other countries. So, the intake of precursors might be higher in other parts of the population^13^. The intake of carotenoid precursors is sufficient for them to be found in human skin, lung and liver, as well as in intestines, prostate and breast^7,14^.

The bioavailability of carotenoids can be affected by food processing, as cell walls are broken down, thereby reducing the binding strength between tissue matrix and carotenoids. Furthermore, consuming food alongside lipids leads to increased solubility of the carotenoids. However, processing can also promote isomerization^15,16^. The bioavailability of the precursors was investigated in an in-vitro simulated gastro-intestinal digestion model, using various fruit juices with coffee cream as a fat source. Phytoene showed with 97% the highest bioavailability closely followed by phytofluene. Mapelli-Brahm et al., 2017, attributed this not only to the higher cis-isomer content but also to the fact that both precursors have fewer conjugated double bonds and therefore exhibit less attraction and aggregation to each other^17^. In contrast, Campbell et al., 2007, showed in an animal experiment with rats, that were fed to 10% with tomato powder for 30 days and then received a single dose of 2.7 mg phytoene or phytofluene, phytofluene accumulated more strongly than phytoene in various tissues (serum, liver, spleen, fatty tissue, testicles) after 24 h. A significant increase could be measured in each case^18^.

An ADI for phytoene and phytofluene does not exist due to the limited data available^19^. In an in-vitro study on cyto- and genotoxicity in rabbit corneal fibroblasts, no significant potential could be measured and short- and long-term in-vivo studies on skin tolerance in humans did not lead to any irritation or sensitization reactions. So, Havas et al., 2018, assumed the topical application as safe. Furthermore, the current phytoene or phytofluene intake is compared to the ADIs of classic carotenoids still far below these concentrations^20^.

Nevertheless, research into the mechanism of action of the precursors alone should be pursued in order to exploit the potential of these carotenoids and the potential of plant varieties such as the tangerine tomato, which are rich in these precursors^21^. For this reason, phytoene and phytofluene were isolated from tomato powder to be able to analyze the effects of the carotenoids on their own. The protective effect against lipid oxidation was analyzed in a model system, the liposomes. The liposomes used consist of uni- and multilamellar membranes and 93% had a size of 197 ± 115 nm, measured by dynamic light scattering (Zetasizer Nano, Malvern Panalytical GmbH, Kassel, Germany). The liposomes represent an established model for investigating lipid oxidation using the biomarker malondialdehyde (MDA)^22,23^. Lipid oxidation was initiated by UV-radiation of different wavelengths. By analyzing UV-C-, UV-B- and UV-A-radiation, the influence of the different mechanisms of action of carotenoids can be considered. Carotenoids can absorb radiation, reduce radicals and scavenge other reactive oxygen species, de-exiting photosensitizers and re-reducing other antioxidants^24^. In UV-irradiated liposomes, only the first two mechanisms can occur. Since phytoene, phytofluene and lycopene absorb different wavelengths, the effect of the antioxidants can possibly be clarified more precisely. The use of UV-radiation to initiate lipid oxidation instead of chemical oxidizing agents is also of great interest, as the protective effects of carotenoids against UV-radiation and consequently skin cancer is still part of research^25^.

## Materials and methods

### Chemicals

The chemicals were either of HPLC-grade quality or analytical-grade and if not otherwise specified from Merck KGaA (Darmstadt, Germany), VWR International GmbH (Darmstadt, Germany), Carl Roth GmbH + Co. KG (Karlsruhe, Germany) or Th. Geyer GmbH & Co. KG (Renningen, Germany). Aqueous solutions were produced with double-distilled water (18 MΩ). The stock solutions of the lipophilic antioxidants as HPLC-standards were dissolved in toluol/cyclohexane (1 + 4, v/v). (*all-E*)-lycopene, was a gift from Conesa (> 99%, Villafranca del Guadiana, Spain), (*all-E*)-*β*-carotene was from Sigma-Aldrich (> 93%, Taufkirchen, Germany), (*all-E*)-phytoene (> 99%), (*E/Z*)-phytofluene (> 99%) and other HPLC-standards and the internal standard for the quantification of antioxidants, *β*-apo-8’-carotenal (> 95%), were from CaroteNature (Münsingen, Switzerland). The concentration of the standards was determined using the Jasco V-530 UV/Vis-Spectrophotometer. (*all-E*)-phytoene and (*E/Z*)-phytofluene were diluted in petroleum ether and measured at 286 nm and 347 nm, (*all-E*)-*β*-carotene at 450 nm in n-hexane and (*all-E*)-lycopene at 470 nm in petroleum ether^26–28^.

### Phytoene and phytofluene-extraction and analysis

Since the carotenoid content increases with dehydration, phytoene and phytofluene were extracted from a tomato powder from sun-ripened tomatoes (gently dried and without additives, Krautschmaus JSD, Ellerbek, Germany)^7^.

The quantification of the carotenoids is described in Heidrich et al.^28^. First, the carotenoids were exhaustingly extracted with MeOH/THF (1 + 1 [v: v] + 0.1% BHT) using an ultrasonic bath. The combined extract solutions were evaporated and redissolved in MeOH/MTBE (1 + 1 [v: v]) and centrifuged (14000 rpm, 5 min) before HPLC analysis^29^.

The carotenoids were analyzed using a VWR Hitachi Chromaster (5000 series) reversed-phase HPLC system (column: Develosil C30, 250 × 4.6 mm, 5 μm, Phenomenex, Aschaffenburg, Germany) at a column temperature of 13 °C and 50 µL injection volume. An eluent gradient method was applied, and the samples were examined at 286 nm for phytoene, 347 nm for phytofluene and 450 nm for the carotenoids with a DAD. For the evaluation, the Chromaster system manager (Version 2.0, Hitachi High-Tech Science Corporation, Tokyo, Japan) was used^28^.

The carotenoid concentrations in the tomato powder used are shown in Table 1.

**Table 1:** Carotenoid concentrations in tomato powder (*n* = 3).

[mg/100 g]	(*all-E*)-Lycopene	(*all-E*)-Phytoene	(*E/Z*)-Phytofluene	(*all-E*)-β-Carotene	(*13Z*)-β-Carotene	(*all-E*)-Lutein	(*all-E*)-Zeaxanthin
Mean value	104	19	7.7	1.56	0.23	0.51	0.12
Standard deviation	17	1	0.2	0.00	0.01	0.06	0.01

(*all-E*)-Lycopene had the highest carotenoid concentration with 104 ± 17 mg/100 g, then (*all-E*)-phytoene followed with 19 ± 1 mg/100 g and (*E/Z*)-phytofluene with 7.7 ± 0.2 mg/100 g. For the extraction of phytoene and phytofluene, 3 g tomato powder were extracted exhaustively, with the same solvent as described above and without an internal standard. The first extraction was made with 30 ml solvent overnight, the second one again with 30 ml solvent for 15 min in the ultrasonic bath and then several extractions, each with 20 ml solvent, until the red color got lighter. The last extractions were only made with 10 ml of the solvent. The combined extracts were evaporated, and the residue was redissolved in MeOH/MTBE (1 + 1 [v: v]). This solution was vacuum filtered several times until no visible supernatant was left on the filter. Then, the extract was further processed using preparative HPLC with UV/Vis-detector (VWR Hitachi Chromaster (7000 series)). Therefore, a reversed-phase column (5 μm, 300 × 10,0 mm, YMC Europe GmbH, Dinslaken, Germany) was used at 24 °C for separation. The injection volume was 1000 µl, and the flow rate was 4 ml/min. A gradient method was applied. It started isocratically for the first 22 min with 85% MeOH and 15% MTBE. In the following 4 min, the MTBE content increased to 25% and then to 27% in 2 min. To clean the column, the MTBE content raised to 60% in 1 min and was held for 2 min before the starting concentration got reset and the method finished after 33 min. Elution of the carotenoids in the tomato powder is shown in Fig. 3.

**Fig. 3:**
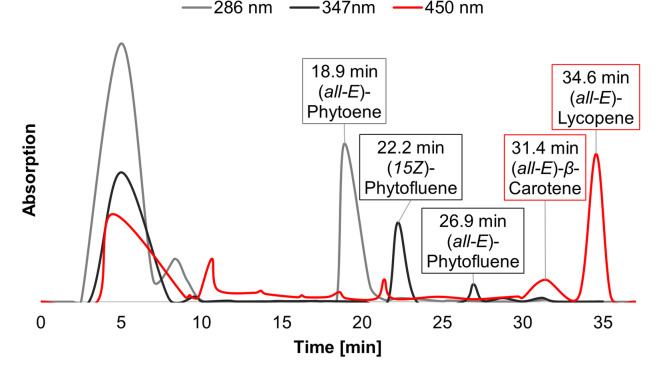
Chromatogram of tomato powder extract on the preparative HPLC.

At the beginning, the UV-detector was set to 286 nm, and after the peak max of phytoene, the wavelength was changed to 347 nm to see when to collect the (*E/Z*)-phytofluene.

The collected fractions were evaporated using a rotary evaporator at 30 °C, and the residue was redissolved in 10 ml dichloromethane. The extracts were checked for purity. The phytoene extract consisted of > 99% (*all-E*)-phytoene and the phytofluene extract of 77% (*E/Z*)-phytofluene and 22% (*all-E*)-phytoene. The phytofluene consisted mainly of (*all-E*)-phytofluene and (*15Z*)-phytofluene^29^.

### Liposome preparation, radiation and quantification of lipid peroxidation: TBA-Assay coupled with HPLC-DAD

The quantification of lipid peroxidation by using the thiobarbituric acid-assay (TBA-Assay) and the production and irradiation of the liposomes is described in Heidrich et al., 2025^28^. The liposome solution, produced from soy lecithin, was irradiated for UV-C-irradiation with 32.4 kJ/m² for 3 min at 254 nm, for UV-B-irradiation with 72.0 kJ/m² for 20 min at 320 nm and for UV-A-irradiation with 144.0 kJ/m² for 30 min at 365 nm in the UV-chamber M3 (Dinies Technologies GmbH, Villingendorf, Germany), in three petri dishes standing on a cooling pad always at the same position. To calculate the influence of the UV-radiation alone, samples were taken from the unirradiated solution and the irradiated solution for the TBA-Assay. MDA was quantified by reversed-phase chromatography using a Merck Hitachi 7000 series HPLC system with a diode-array-detector (DAD). The column (Luna^®^ C18, 250 × 4.6 mm, 5 μm, Phenomenex, Aschaffenburg, Germany) temperature was 25 °C, and the elution was isocratic with a mixture of potassium-phosphate buffer (pH = 5.5)/MeOH (55:45 [v: v]). Each day, a 6-point calibration curve in the range 0.21–4.26 mg MDA/ml (*r* > 0.999) was measured^28^.

### Limits of detection and quantification

The signal-to‐noise ratios of S/*N* = 3:1 and S/*N* = 10:1 were used for the limits of detection and quantification for the antioxidants, and the DIN 32,645 was used for the MDA.

### Statistical analysis

To calculate the significance of the results, IBM^®^ SPSS^®^ Statistics (Version 31.0.0.0, Chicago, IL, USA) was used. Via the Levene’s test (*p* > 0.05), the homogeneity of variances was evaluated. One-factor ANOVA and Bonferroni or Games-Howell post hoc test (α = 0.05) were applied. The latter was used, if the homogeneity of variances was not given. The Bonferroni correction was applied because it is very conservative. This prevents the assumption of a significant effect although none exists. The results are presented as mean ± standard deviation.

## Results and discussion

### Protective effects of colorless carotenoids in comparison to lycopene in UV-irradiated liposomes

The calculation of the protective effect was made by subtracting the MDA concentration of the sample from the control sample (without antioxidants), and setting the result in relation to the control sample^28^:

$$\:Protective\:Effect=\frac{\mathrm{c}\mathrm{o}\mathrm{n}\mathrm{t}\mathrm{r}\mathrm{o}\mathrm{l}\:\mathrm{s}\mathrm{a}\mathrm{m}\mathrm{p}\mathrm{l}\mathrm{e}-\mathrm{s}\mathrm{a}\mathrm{m}\mathrm{p}\mathrm{l}\mathrm{e}}{control\:sample}\cdot\:100$$

**Fig. 4:**
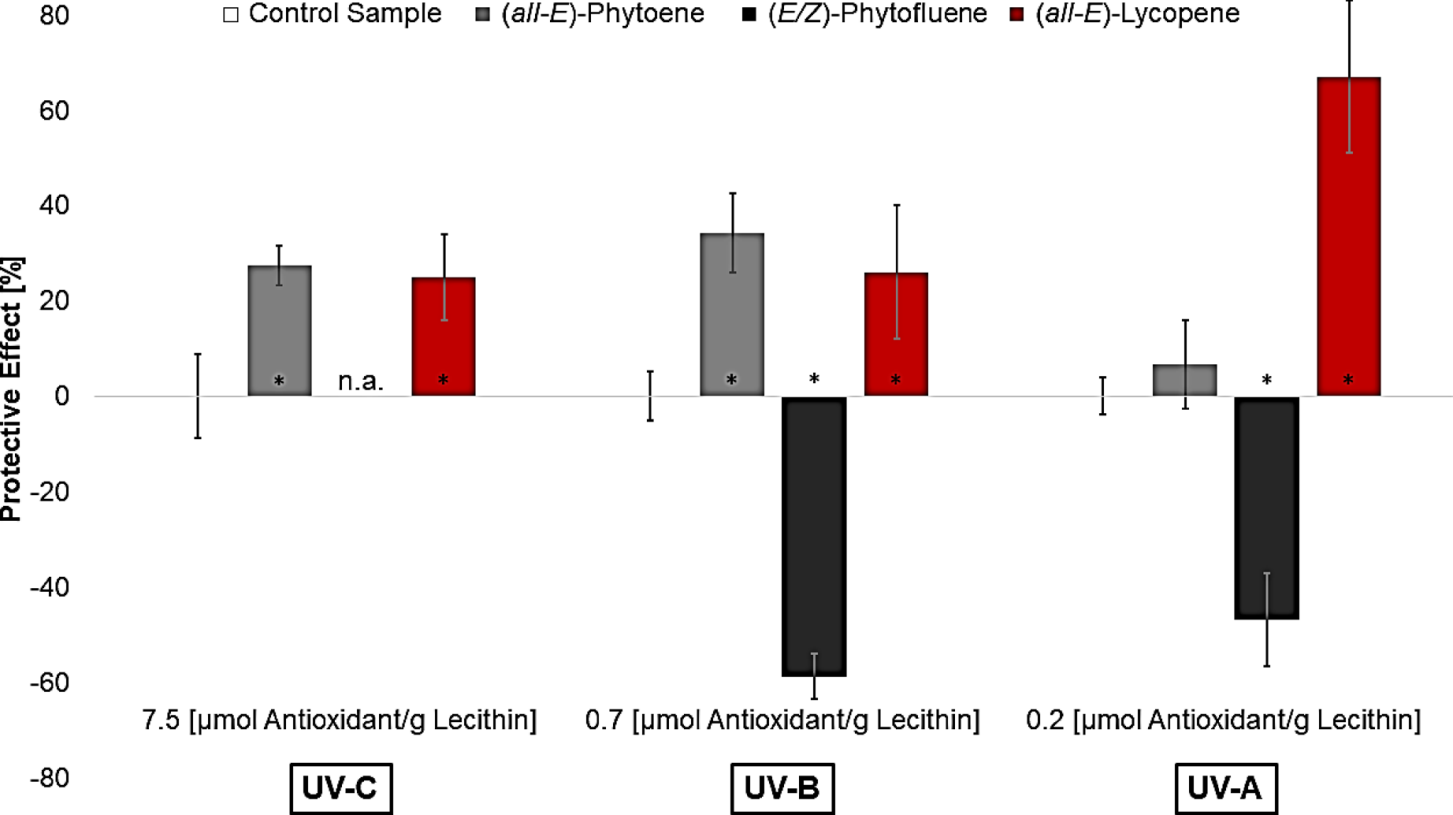
Protective effects of the colorless carotenoids and lycopene against UV-induced lipid oxidation in liposomes tested at concentrations with the first significant protective effect of lycopene at each wavelength, n.a.= not analyzed; * = significantly different to the control sample, (*n* = 9; p-value < 0.05)^28^.

In Fig. 4, the protective effects of phytoene and phytofluene are shown and compared to the protective effect of lycopene (data from^28^). The protective effect of phytofluene against UV-C-radiation was not analyzed since huge concentrations were needed. The chosen concentration for each wavelength was dependent on the lycopene concentration which had the first significant protective effect against the wavelength in the study of Heidrich et al., 2025^28^.

Phytoene showed a significant protective effect of 27% under UV-C-radiation, which is almost equal to that of lycopene (25%). In UV-B-irradiated liposomes, phytoene protected against lipid oxidation even better (34%) than lycopene (26%). However, the difference is not significant. Phytoene had no protective effect against UV-A-radiation, whereas lycopene achieved the strongest protection with 67%. A comparison of the absorption spectra of phytoene and lycopene (Fig. 2) shows that phytoene absorbs parts of the UV-C- and UV-B-radiation and lycopene absorbs only a little of UV-A-radiation. In addition, phytoene and lycopene only differ in their number of double bonds, especially conjugated double bonds. Phytoene has nine double bonds of which three are conjugated and lycopene has thirteen double bonds of which eleven are conjugated. This means that radicals are more strongly resonance-stabilized by lycopene than by phytoene^30^. Edge and Truscott also showed this effect for carotenoids with a smaller difference in the number of conjugated double bonds^30^. The strong protective effect of lycopene under UV-A-radiation and the still significant effects under UV-C- and UV-B-radiation are probably due to the excellent π-electron system, but also due to the pure absorption of radiation. This thesis is supported by phytoene, which is less able to resonance-stabilize radicals due to the few conjugated double bonds. Thus, phytoene cannot reduce the lipid oxidation caused by UV-A-radiation. As phytoene had a similar effect to lycopene under UV-C- and UV-B-radiation, although it is less effective at scavenging radicals, the pure absorption of UV-radiation plays a significant role in the protective effect of carotenoids. The absorption maximum of phytofluene is between those of phytoene and lycopene. So phytofluene absorbs mainly UV-B- and UV-A-radiation. In addition, phytofluene has five conjugated double bonds of ten double bonds in total. Martinez et al., 2014, analyzed the antioxidants using the Trolox Equivalent Antioxidant Capacity Test (TEAC test). They showed that lycopene had the highest activity with 3.52 mM Trolox, followed by phytofluene with 2.57 mM Trolox and finally phytoene achieved the lowest result with 1.02 mM Trolox. The order was expected due to the small energy difference between the triplet and singlet state. Nevertheless, the TEAC test-values for phytoene and phytofluene were comparatively high^31^. Accordingly, a protective effect was also expected for phytofluene at the tested wavelengths. However, the MDA content in the liposomes increased in both cases. Consequently, a significant prooxidative effect was measured at both wavelengths.

###  Antioxidant-degradation of the colorless carotenoids in comparison to lycopene in UV-irradiated liposomes

**Fig. 5:**
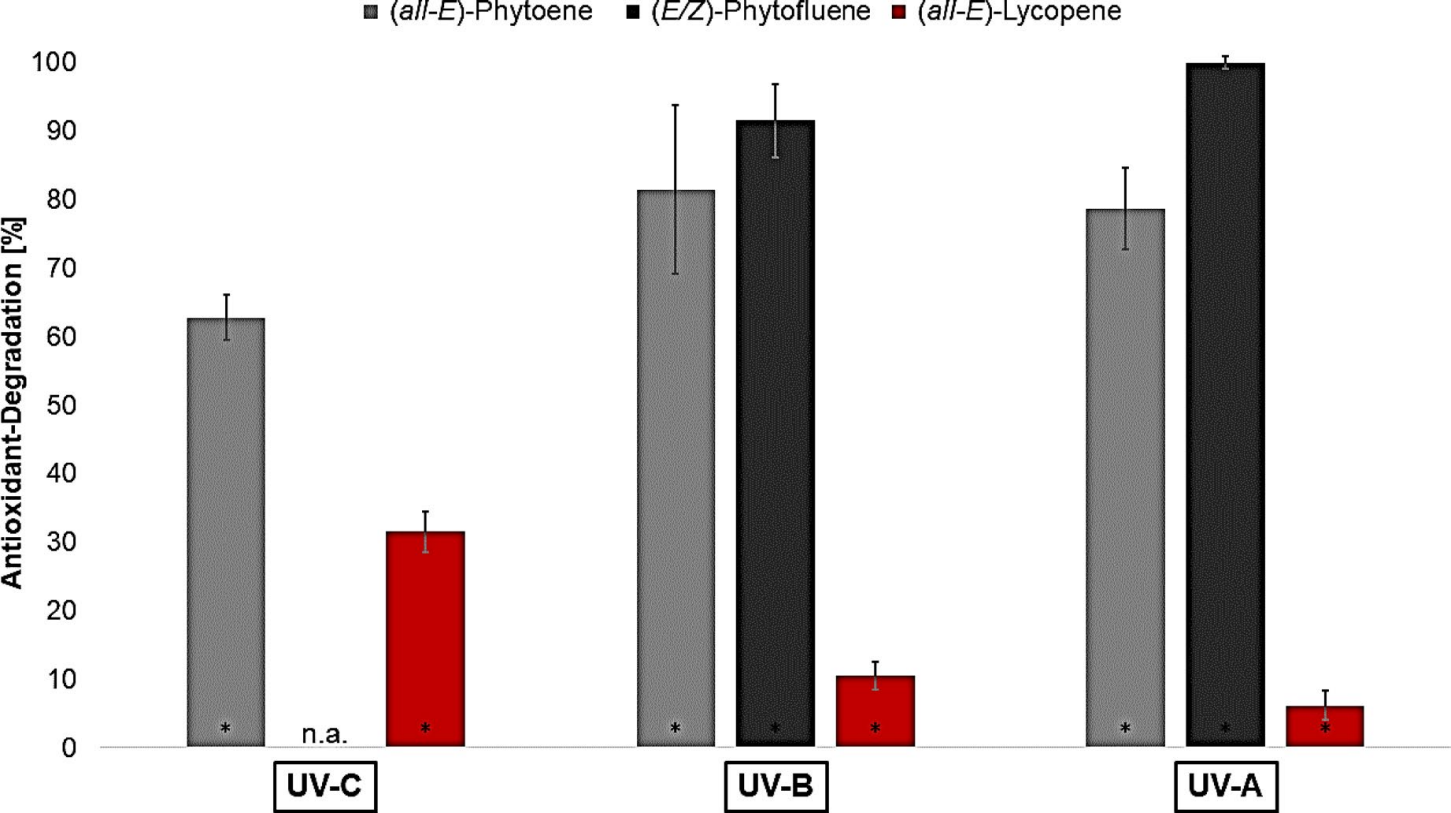
Antioxidant-Degradation of the colorless carotenoids and lycopene tested in UV-irradiated liposomes with an antioxidant concentration of 2 µmol/g lecithin, * = significantly different to the antioxidant-concentration before irradiation, n.a.= not analyzed (*n* = 3; p-value < 0.05)^28^.

To gain further insight into the effects of the carotenoids, the degradation of the antioxidants was analyzed (Fig. 5).

**Fig. 6:**
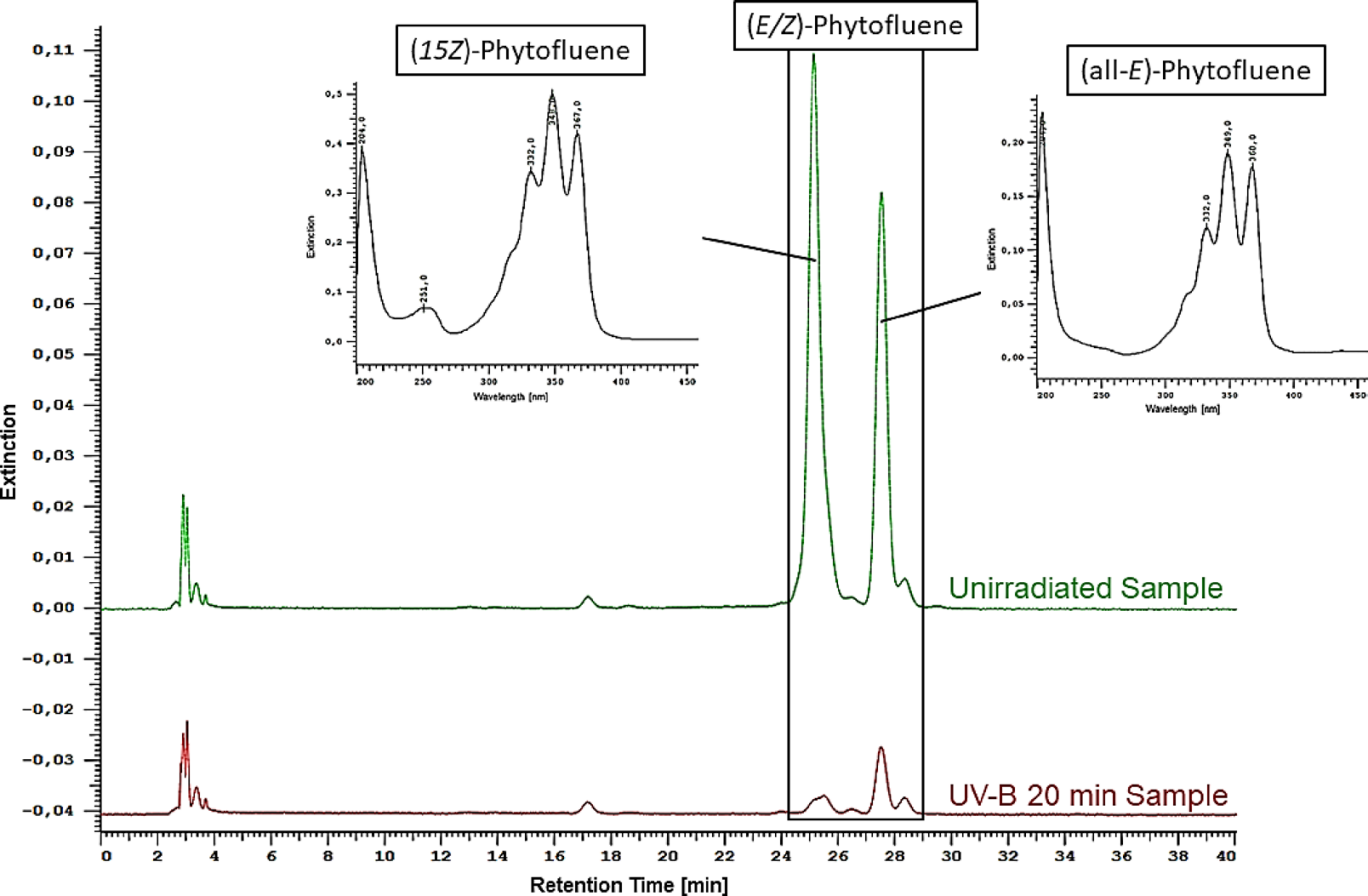
Chromatogram of an unirradiated sample containing (*E/Z*)-phytofluene and the same sample after 20 min of irradiation with UV-B, UV/Vis-Spectrum on the left belonging to (*15Z*)-phytofluene and on the right to (*all-E*)-phytofluene.

First, it is noticeable that the colorless carotenoids were more strongly degraded and therefore less stable than lycopene. Phytoene degraded by 63% under UV-C-radiation, 81% under UV-B- and 79% under UV-A-radiation. Phytofluene degraded by 92% under UV-B-radiation and under UV-A-radiation the final concentration of phytofluene was below the detection limit. The most important factors regarding antioxidant degradation are heat, light, matrix and oxygen^9,32^. Since the matrix is the same in all experiments and generation of heat was prevented by cooling the samples, the irradiation in particular had the greatest influence during the radiation^32^. It should be noted that UV-C-irradiation was much shorter (3 min) than UV-B- (20 min) and UV-A-irradiation (30 min). This means that the antioxidants are exposed to oxygen for a much longer period during the radiation with longer wavelength. Phytofluene in particular is said to be very unstable in the presence of oxygen, which can partly explain the strong degradation^26^. In addition, different protective effects of carotenoids, especially lycopene, have also been recognized depending on the oxygen partial pressure^33–35^. Since the experiments were not done at different oxygen partial pressures, differences and dependencies cannot be considered in this respect. Furthermore, the phytofluene-extract did not consist of pure (*all-E*)-phytofluene. As can be seen in Fig. 6, there are mainly two isomers present in the phytofluene-extract.

**Fig. 7:**
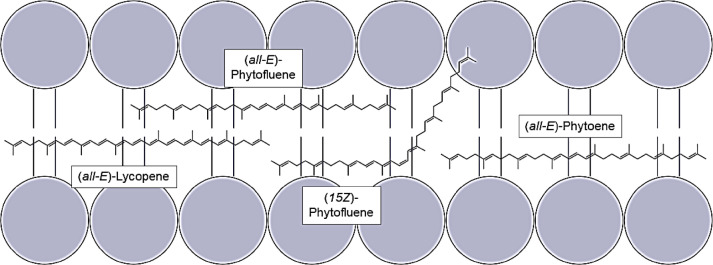
Localization of the analyzed carotenoids in the lipid bilayer.

The large first peak corresponds to (*15Z*)-phytofluene, due to the “cis-peak” at 251 nm and to the fact that this cis-isomer occurs more frequently in tomatoes than others^10,36^. The second major peak corresponds to (*all-E*)-phytofluene. The chromatogram of the sample irradiated with UV-B-radiation demonstrates that the cis-isomer is less stable than the (*all-E*)-phytofluene. Shi et al., 2002, showed the same correlation for lycopene-isomers^37^.This knowledge provides insights into the observed pro-oxidative effect of phytofluene. Finally, (*15Z*)-phytofluene has a kink in the molecule near the center. This kink in the molecule could cause that it does not fit in the non-polar part of the lipid bilayer of the liposomes (Fig. 7).

The lipid bilayer consists of phospholipids. The non-polar fatty acids face inwards, so that the interior of the lipid bilayer is non-polar and the edge is more polar due to the phosphate groups. As the three carotenoids studied are highly non-polar, they are located within the membrane^38^. However, a kink in the molecule could cause that the (*15Z*)-phytofluene sticks out of the lipid bilayer on one side. This allows radicals in the aqueous phase to be captured and to enter the interior of the membrane. Carotenoid radicals can also lead to a pro-oxidative effect under certain circumstances^33^. (*15Z*)-Phytofluene is one example for that kind of radical transfer bridge which significantly increases the concentration of radicals in the lipid bilayer^39^. Another reason is that cis-isomers are less stable than their (*all-E*)-isomers. For lycopene, Srivastava and Srivastava showed that (*all-E*)-lycopene was 30% more stable under a daylight lamp than cis-isomers of lycopene^32^. In addition to the higher instability of cis-isomers, the quenching potentials of (*all-E*)-isomers, measured in benzene, are greater than those of the corresponding cis-isomers^35^. Consequently, considering the isomer pattern of phytofluene, a low protective effect could be assumed.

## Conclusion

In summary, the developed method can be used to isolate phytoene and phytofluene from tomato extract. But only phytoene had a protective effect against UV-C- and UV-B-radiation. As there was no effect against UV-A-radiation, it can be assumed that the pure light absorption of carotenoids is not negligible compared to the effect of lycopene. The lack of an antioxidant effect of the phytofluene extract was not initially expected because it contained around 22% phytoene. However, the isomer pattern and thus the high proportion of (*15Z*)-phytofluene, naturally given by the tomato, probably led to these results. The mechanism of the effect of (*15Z*)-phytofluene should be investigated further alone and in mixtures in human studies to confirm this statement. If they support the thesis, previous statements found in the literature that phytoene and phytofluene in plant extracts have led to a better antioxidant effect than lycopene alone should be re-examined.

## Data Availability

The original contributions presented in this study are included in the article. Further inquiries can be directed to the corresponding author.

## Abbreviations

DAD: Diode array-detector
HPLC: High-pressure liquid chromatography
MDA: Malondialdehyde
MeOH: Methanol
MED: Minimal erythema dose
MTBE: Methyl tert-butyl ether
UV: Ultraviolet
Vis: Visible light
TBA: Thiobarbituric acid
TEAC: Trolox Equivalent Antioxidative Capacity
THF: Tetrahydrofuran
